# Effect of Religiosity and Dysfunctional Dating Attitudes on Youth Substance Use

**DOI:** 10.1155/2014/143709

**Published:** 2014-08-05

**Authors:** Andra Teten Tharp, C. Nathan DeWall, Stephanie B. Richman, Rita K. Noonan

**Affiliations:** ^1^Centers for Disease Control and Prevention, 4770 Buford Highway, MS F-64, Atlanta, GA 30341, USA; ^2^University of Kentucky, Lexington, KY 40506, USA

## Abstract

The current investigation examined the interactive effect of dysfunctional dating attitudes and religiosity on substance use in a large sample of youth (*N* = 1,357) from the *YouthStyles* survey. Based on past research, we explored the possibility that religiosity buffered the association between dysfunctional dating attitudes and substance use. Because age was significantly associated with all study variables, we included age in our analyses. In support of our hypothesis we found an attitude by religiosity by age interaction among youth with moderate levels of dysfunctional dating attitudes. Among these youth, the buffering effect of religiosity increased with age. For youth with low and high dysfunctional dating attitudes, religiosity did not buffer the association. The results of this study are in line with past work that suggests that the association between relationship characteristics and substance use is complex. It also identifies religiosity as a protective factor for the effect of dating attitudes on substance use but suggests that these effects may be the most important for youth with moderate levels of dysfunctional dating attitudes.

## 1. Introduction

Substance use and relationship problems are significant issues facing youth. The 2013 Youth Risk Behavior Survey found that 34.9% of high school students had used alcohol and 23% used marijuana in the 30 days prior to the survey; among the 73.9% of high school students who had dated in the 12 months prior to the survey, 10.3% experienced prior-year physical dating violence and 10.4% experienced prior-year sexual dating violence [1]. Substance use has been supported as a risk factor for dating violence perpetration and victimization (for review, see [2]) and as a consequence of dating violence victimization [3].

Less work has examined the role that dating attitudes and relationship characteristics play in youth's substance use. Using data from the National Longitudinal Study of Adolescent Health, two studies have found that partner and relationship characteristics can have differing effects on subsequent substance use. Kreager and Haynie [4] found that, for boys and girls who reported that the friends of their dating partner used alcohol, their own drinking frequency and binge drinking had increased when assessed one year later. In contrast, Gudonis-Miller, et al. [5] found that as relationship seriousness increased over time, marijuana use decreased. Taken together, these studies suggest that aspects of youth romantic relationships can be either risk or protective factors for substance use.

While the research on youth relationships grows, adult literature suggests that the effects of relationship characteristics on substance use may be salient only at certain levels of the risk factor. For example in one study, antisocial personality disorder (ASPD) moderated the association between alcohol consumption and intimate partner violence (IPV; see [6]). Alcohol consumption was associated with an increased likelihood of nonsevere IPV among men without ASPD but not among men with ASPD. Instead, these men were likely to engage in nonsevere IPV regardless of whether or not they drank, but drinking was more strongly associated with a likelihood of severe IPV among men with ASPD [6]. This study suggests that individuals may have a threshold for certain relationship characteristics and the threshold may be temporarily increased or decreased based on the presence of another factor. For example, individuals may be at increased risk when their individual threshold is exceeded, and alcohol consumption is one factor that may lower this threshold [7]; however, alcohol consumption will have little impact on individuals who are above or far below the threshold, as lowering it would have not change their relative position to the threshold. People who are above their threshold will be at increased risk whether or not they have consumed alcohol, and people far below their threshold will not increase enough to rise above their threshold.

Extending this model, religiosity may alter the threshold at which different relationship characteristics have an impact on substance use. Religiosity has consistently been associated with lower levels of substance use among youth [8, 9], and religiosity and abstaining from alcohol have been associated with attitudes that support sexual abstinence [10], suggesting that attitudes about dating and sex, religiosity, and substance use are associated among youth. In line with social control theory [11], religious youth may choose not to engage in risky or illegal behaviors, such as early sexual debut or substance use, as a function of their commitment to the values espoused by their faith and the attachment to the people and institutions that are aligned with their religious beliefs. For example, many religions do not support sexual activity and substance use among youth and as such youth who affiliate with these religions may be less likely to engage in these risky behaviors or endorse beliefs supportive of such behaviors under certain circumstances.

However, individual differences would suggest that the effect of religiosity on attitudes and engagement in risky behaviors is not uniform, but the specific circumstances or factors with which religiosity may have protective effects are less clear. Religious individuals may have a higher threshold than nonreligious individuals (as noted with ASPD above). This would suggest that at low levels of unhealthy relationship characteristics, such as holding dysfunctional dating attitudes, religious individuals will be as likely to engage in substance use as nonreligious individuals because the risk factor (i.e., dysfunctional dating attitudes) has not exceeded minimum threshold levels to have an impact on substance use. At moderate levels of dysfunctional dating attitudes, religious individuals will be less likely to use substances than nonreligious individuals. At higher levels of dysfunctional dating attitudes, religious individuals may be just as likely to engage in substance use as nonreligious individuals because the amount of dysfunctional dating attitude is so elevated that it lowers the threshold enough for them to engage in substance use.

The current study takes a first step in exploring the interactive association of religiosity and youth dysfunctional dating attitudes with substance use. It was expected that dysfunctional dating attitudes and substance use would be significantly correlated and that religious youth would report significantly lower levels of substance use and dysfunctional dating attitudes than nonreligious youth. Finally, it was expected that, among youth with moderate levels of dysfunctional dating attitudes, religiosity would interact with dysfunctional dating attitudes in its prediction of youth substance use, such that the association between attitudes and substance use is significantly less robust among youth who report that religion is an important part of their life. We did not expect such an association among youth with low and high levels of dysfunctional dating attitudes. This research is critical to build the understanding of relationship factors that may influence substance use among youth, capture the nuance implicit in youth risk behaviors, and identify potential protective factors that can serve as the basis of health promotion and prevention strategies.

## 2. Method

*Participants.* Participants were the 1,357 respondents to the 2007* YouthStyles* survey. Participant characteristics are described in Table 1.

*Procedure.* The* YouthStyles* survey is part of Styles 2007, which is comprised of three consumer mail panel surveys,* ConsumerStyles*,* YouthStyles*,* and HealthStyles*, administered in two waves. The sampling and data collection for Styles 2007 were conducted by Synovate, Inc. Respondents were recruited to join the mail panel through a four-page recruitment survey. In return for their participation, respondents were given a small incentive and were entered into a sweepstakes. For the initial wave, stratified random sampling was used to generate a list of 20,000 potential respondents. A “households-with-children” supplement (*N* = 6, 000) was used to ensure adequate numbers of potential respondents for the* YouthStyles* survey during the second wave. In 2007, the response rate for the households-with-children supplement was 58.1%. In the second wave, 2,566* YouthStyles* surveys were sent to half of the mail panel households that returned the* ConsumerStyles* survey for the initial wave (*N* = 11, 758). Youth and parents used separate postage-paid return envelopes. Responses were received from 1,357* YouthStyles* participants, yielding a response rate of 52.8%.

*Instruments.* Youth responses to the attitudes and opinions section of* YouthStyles* were used in the current study.

Religiosity was assessed using the following item:* religion is an important part of my life*. Youth responded to this item on a 4-point Likert-type scale, on which 1 =* Really Disagree* and 4 =* Really Agree*. For data analysis, dichotomous responses were created from the religiosity responses, such that* Really Agree *and* Agree* were both coded as “Agree” and* Really Disagree* and* Disagree* were both coded as “Disagree.”

As in past work [12], to assess attitudes supporting controlling dating relationships (referred to here as dysfunctional dating attitudes), we used the following items:* My idea of a good relationship is having a boyfriend/girlfriend who keeps track of me at all times, My idea of a good relationship is having a boyfriend/girlfriend who gets jealous when I talk to other boys/girls*, and* My idea of a good relationship is having a boyfriend/girlfriend who spends all of his/her free time with me*. Youth responded to these items on a 4-point Likert-type scale, on which 1 =* Really Disagree* and 4 =* Really Agree*. For primary data analysis, item responses were summed to obtain a total score for dysfunctional dating attitudes (possible range 3–12). Cronbach's alpha for the three items was 0.64. Because we expected effects at moderate but not low or high levels of dysfunctional dating attitudes, this variable was trichotomized. Total scores between 3 and 5 were considered low dysfunctional dating attitudes, scores between 6 and 9 were considered moderate dysfunctional dating attitudes, and scores between 10 and 12 were considered high dysfunctional dating attitudes.

To assess substance use, we selected items that assessed use of tobacco, alcohol, and marijuana. In response to each item, youth indicated whether they* “Never used (the substance)”* (coded 1),* “Tried (the substance), but did not use in the past month”* (coded 2), or* “Used (the substance) in the past month”* (coded 3). Item responses were summed to obtain a total score for substance use. Cronbach's alpha for the three items was 0.87.

## 3. Results

Frequency of religiosity and means and standard deviations for age, substance use, and dysfunctional dating attitudes for the sample are reported in Table 1. Of the study participants, eight hundred seventy-nine (64.8%) reported that religion was important to them. Substance use and dysfunctional dating attitudes had means of 3.79 (SD = 1.37) and 5.52 (SD = 1.96), respectively. Correlations are shown in Table 2.

As expected, dysfunctional dating attitudes were significantly correlated with substance use, *r* = 0.10, *p* < 0.01. Religiosity was negatively associated with substance use, *r*_*pb*_ = − 0.17, *p* < 0.01, and was not significantly correlated with dating attitudes. Age was also significantly associated with dysfunctional dating attitudes, *r* = 0.07, *p* < 0.05, religiosity, *r*_*pb*_ = − 0.08, *p* < 0.01, and substance use, *r* = 0.37, *p* < 0.001, indicating that dysfunctional dating attitudes and substance use increased with age and religiosity decreased with age.

Two one-way ANOVAs were used to examine mean differences in dysfunctional dating attitudes and substance use between religious and nonreligious youth. No significant mean difference on dysfunctional dating attitudes between nonreligious (*M* = 5.41, SD = 1.89) and religious (*M* = 5.57, SD = 1.99) youth was found, *F*(1,1254) = 1.89, *p* = 0.17. Consistent with prior work, religious youth (*M* = 3.62, SD = 1.21) reported significantly less substance use than nonreligious youth did (*M* = 4.13, SD = 1.60), *F*(1,1270) = 39.08, *p* < 0.001.

Although we had intended to examine only the interaction between dysfunction dating attitudes and religiosity, age had significant bivariate correlations with these variables. Therefore, we included age as a main effect and in two- and three-way interactions in the model. Gender was not included in the regression because it did not have significant associations with any study variable at the bivariate level. To determine if religiosity buffered dysfunctional dating attitudes in its prediction of youth substance use, we conducted a linear regression with substance use as the dependent variable and dysfunctional dating attitudes, religiosity, age, attitude by religiosity, age by attitude, age by religiosity, and attitude by religiosity by age interactions as the independent variables. We performed three regressions to explore associations among the variables at low, moderate, or high levels of dysfunctional dating attitudes. Results are presented in Table 3. At low levels of dysfunctional dating attitudes, age was significant. At moderate levels of dysfunctional dating attitudes, attitudes, the attitude by religiosity, attitude by age, religiosity by age, and religiosity by attitude by age interactions were significant. At high levels of dysfunctional dating attitudes no effects were significant. The religiosity by age interaction suggests that religiosity buffers the age-related increase in substance use. The attitude by age interaction suggests that dysfunctional dating attitudes are increasingly associated with substance use as youth age. The attitude by religiosity interaction suggests that religiosity buffers the association between dysfunctional dating attitudes and substance use. The results of the three-way interaction suggest that, for youth with moderate levels of dysfunctional dating attitudes, the buffering effect of religiosity was stronger for older compared to younger youth.

**Figure 1:**
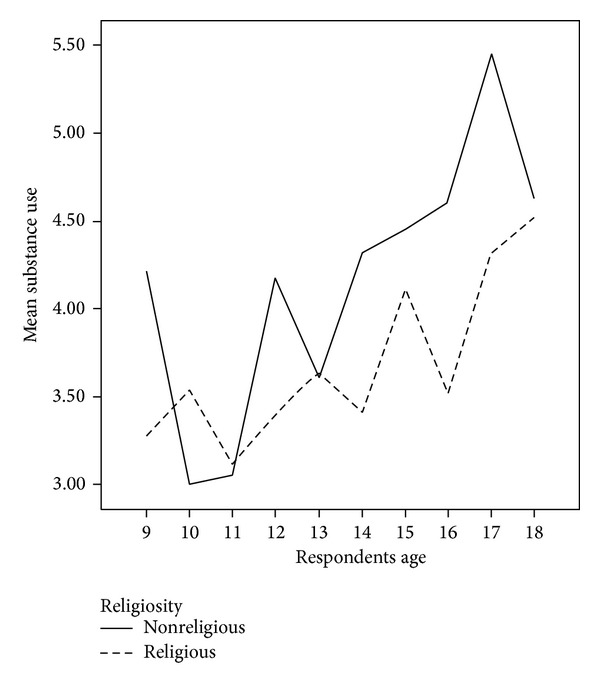
Effects of dysfunctional dating attitudes, religiosity, and age on substance use for youth with moderate levels of dysfunctional dating attitudes. Cases weighted by* YouthStyles* weighting variable.

These findings support our prediction that religiosity will exert a buffering effect on substance use primarily at moderate levels of dysfunctional dating attitudes and for youth that have a substance use history that extends beyond mere experimentation that is often found among younger youth. In contrast, for youth with low and high dysfunctional dating attitudes religiosity did not buffer the association. Again, these findings confirm our hypothesis that once individuals have exceeded a certain threshold of dysfunctional dating attitudes, religiosity will no longer exert a buffering effect on their substance use. The effects of dysfunctional dating attitudes, religiosity, and age on substance use for youth with moderate levels of dysfunctional dating attitudes are presented in Figure 1.

## 4. Discussion

The prevalence and consequences of youth risk behaviors, such as substance use and unhealthy relationships, underscore the need to better understand and prevent these issues. However, little work has examined the effect of relationship characteristics, other than violence, on substance use. The complexity of youth relationships suggests that investigations into this area capture the nuance of these factors, for example, by examining how associations vary by level of a risk factor and by age. Towards this end, the current investigation examined the interactive effect of dysfunctional dating attitudes, religiosity, and age on substance use in a large sample of youth. Among youth with moderate levels of dysfunctional dating attitudes, we found a main effect for attitudes, two-way interactions for attitudes by religiosity, age by religiosity, and age by attitudes, as well as a three-way interaction for attitudes by religiosity by age. Further investigation of this three-way interaction suggested that religiosity buffered the association between dysfunctional dating attitudes and substance use for youth with moderate levels of dysfunctional attitudes and that this buffering effect was stronger for older compared to younger youth. In contrast, for youth with low and high dysfunctional dating attitudes, religiosity did not buffer the association between attitudes and substance use. Thus, the current investigation extends past work that demonstrated that relationship characteristics influence youth's substance use [4, 5] and suggests that religiosity can have a nuanced role as a protective factor against substance use. For example, similar to Gudonis-Miller et al. [5], who found that as relationship seriousness increased over time marijuana use decreased, the current study found that the buffering effect of religiosity on the association between dysfunctional dating attitudes and substance use was more pronounced for older compared to younger youth.

Our findings add to a body of literature suggesting that protective factors have variable effects under different conditions. For example, “loss of face,” a belief that one's actions are a reflection on one's family, friends, and community, has been shown to be a protective factor for sexual violence in the context of certain risk factors and in certain ethnic groups [13]. Furthermore, the finding that religiosity buffered the effect of dysfunctional dating attitudes at certain ages and at certain levels of dysfunctional dating attitudes is consistent with past work showing that protective factors may be most salient at moderate levels of a risk factor [6], presumably because this is when risk factors begin to exert their effect on an outcome measure. In light of social control theory, loss of face and religiosity may function similarly as protective factors because both represent attachment to people, institutions, or values that may inhibit youth engagement in risky or illegal behaviors. However, the current study extends past work that has primarily demonstrated direct associations and found that the protective effect was only viable at moderate levels of dysfunctional dating attitudes. The implication is that a protective factor, such as religiosity, exerts its strongest impact when a person's dysfunctional dating attitudes are on the verge of placing them at risk for engaging in frequent substance use behaviors. Once a person's dysfunctional dating attitudes have extended to a high level, it is difficult for religiosity (and potentially other protective factors) to buffer them from the risk of engaging in frequent substance use behaviors.

The study was limited by several factors. First, the sample was cross-sectional and was not nationally representative. Specifically, as noted in Tharp and Noonan [12], the rates of substance use in the* YouthStyles* sample appear to be lower than national averages. Second, the assessment of religiosity, dysfunctional dating attitudes, and substance use was self-report and was based on the* YouthStyles* survey questions, rather than validated instruments. Despite this limitation, most scales had reasonable internal consistency coefficients and the assessment of religiosity was similar to what is used on other studies, although most studies ask about other characteristics of religiosity in addition to self-rating of the importance of religion. Although our sample size provided a well-powered test of our hypotheses, these limitations suggest that the results should be viewed as preliminary and in need of replication. That said, the identification of viable protective factors is needed and the current study is a first step in examining the nuanced interaction of risk and protective factors that may contribute to youth substance use.

Research examining nuanced associations between youth risk behaviors is needed to inform the development and refinement of health promotion and prevention strategies. As the field of public health shifts from a focus on disease prevention to one of health promotion, the identification of protective factors is particularly critical. The current study extended past work on the effects of relationship characteristics on substance use by examining how religiosity buffered this association and how this association varied by age. Results suggest that religiosity may be more salient as a protective factor for older youth. Bivariate correlations suggested that in general religiosity was lower for older compared to younger youth, so this finding suggests that, for youth who continue to report that religion is an important part of their life, the protective effects have increased salience over time. Based on these findings, religious organizations may identify additional methods to keep youth engaged as youth age and prevention approaches may integrate spirituality with other positive youth development approaches.

## Figures and Tables

**Table 1:** Participant characteristics (*N* = 1357).

Characteristic	*N* (%)
Gender	
Boys	693 (51.1%)
Girls	664 (48.9%)
Age (*M* (SD))	13.44 (2.81)
Parent's marital status	
Currently married	1032 (76%)
Divorced	130 (9.6%)
Never married	102 (7.5%)
In domestic partnership	55 (4.1%)
Separated	20 (1.5%)
Widowed	15 (1.1%)
Religiosity	
Nonreligious	445 (32.8%)
Religious	879 (64.8%)
Substance use (*M* (SD))	3.79 (1.37)
Dysfunctional dating attitudes (*M* (SD))	5.52 (1.96)

**Table 2:** Correlations among participant age, gender, dysfunctional dating attitudes, religiosity, and substance use.

	Gender *r*_*pb*_	Age *r*	DDA *r*	Religiosity *r*_*pb*_
Gender				
Age	0.01			
Dysfunctional dating attitudes (DDA)	−0.04	0.07∗		
Religiosity	0.01	−0.08∗∗	0.04	
Substance use	0.02	0.37∗∗∗	0.10∗∗∗	−0.17∗∗∗

*p < .05. **p < .01. ***p < .001.

**Table 3:** Regressions examining the associations between dysfunctional dating attitudes, religiosity, age, and youth substance use.

	Low DDA *β*	Moderate DDA *β*	High DDA *β*
*n*	641	528	38

*F*	24.09∗∗∗	12.73∗∗∗	4.35∗∗

Dysfunctional dating attitudes (DDA)	0.05	−1.30∗	−3.51
Religiosity	0.91	−9.53	−22.20
Age	0.27∗	−0.55	−3.23
DDA × religiosity	0.02	1.55∗	2.51
DDA × age	−0.004	0.12∗∗	0.35
Religiosity × age	−0.09	0.80∗	2.01
Religiosity × DDA × age	−0.001	−0.13∗∗	−0.22

*p < .05. **p < .01. ***p < .001.
